# Effects of *Acremonium terricola* Culture on the Growth, Slaughter Yield, Immune Organ, Serum Biochemical Indexes, and Antioxidant Indexes of Geese

**DOI:** 10.3390/ani12091164

**Published:** 2022-05-02

**Authors:** Jinyuan Chen, Yawen Guo, Yang Lu, Zhaoyuan He, Yali Zhu, Shuyu Liu, Kaizhou Xie

**Affiliations:** 1 College of Animal Science and Technology, Yangzhou University, Yangzhou 225009, China; mz120191044@yzu.edu.cn (J.C.); dx120200135@yzu.edu.cn (Y.G.); mz120191020@yzu.edu.cn (Y.L.); mz120191027@yzu.edu.cn (Z.H.); mz120211463@stu.yzu.edu.cn (Y.Z.); mz120211479@stu.yzu.edu.cn (S.L.); 2 Joint International Research Laboratory of Agriculture and Agri-Product Safety, Yangzhou University, Yangzhou 225009, China

**Keywords:** *Acremonium terricola* culture, growth performance, slaughter yield, antioxidant function

## Abstract

**Simple Summary:**

The culture of *Acremonium terricola* has similar components to *Cordyceps* and exhibits growth-promoting and antioxidant effects. This research investigated the effects of *Acremonium terricola* culture on the growth performance, slaughter performance, immune organ and serum biochemical indexes, and serum and muscle antioxidant indexes of geese. The results showed that adding *Acremonium terricola* culture to the diet could reduce the feed-to-weight ratio of geese and improve the antioxidant capacity of goose meat and serum. The results indicated that *Acremonium terricola* culture could be used as a new feed additive to promote the growth of geese and improve the antioxidant capacity of geese.

**Abstract:**

*Acremonium terricola* culture (ATC) is a new type of green feed additive, and its main components include cordycepin, adenosine, and ergosterol. In this study, the Hortobagy geese were used as the experimental animals to explore the effects of ATC addition to the basal diet. Seven hundred and twenty 1-day-old Hortobagy geese were randomly divided into four treatment groups, each with 180 geese divided into six pens equally. The four treatments included the control group and three experimental treatments. Half of the geese in each group were males and half were females. All geese were offered the same basal diet with ATC supplementation at 0, 3, 5, and 7 g/kg. The results showed that basal diet supplementation with 7 g/kg ATC reduced the feed conversion rate (FCR) of Hortobagy geese in a highly significant manner (*p* < 0.01). When the dosage of ATC was 3 g/kg, the breast muscle rate and leg muscle rate of female geese were significantly increased (*p* < 0.05). ATC supplementation in the basal diet had no significant effect on the immune organ index of Hortobagy geese (*p* > 0.05). Basal diet supplementation with 3 g/kg and 5 g/kg ATC significantly reduced the alanine aminotransferase (ALT) content in the serum of female geese, significantly increased the total antioxidant capacity (T-AOC) of the serum, and significantly reduced the malondialdehyde (MDA) content in the serum (*p* < 0.05). The addition of 5 g/kg and 7 g/kg ATC to the basal diet reduced the blood glucose (GLU) content in male geese in a highly significant manner (*p* < 0.01). A basal diet supplemented with 3 g/kg and 7 g/kg ATC significantly reduced the MDA content in geese breast muscles (*p* < 0.05). Basal diet supplementation with 3 g/kg ATC highly significantly improved the T-AOC of female geese breast muscles (*p* < 0.01). Basal diet supplementation with 5 g/kg ATC significantly improved the T-AOC of female geese leg muscles (*p* < 0.01). In summary, basal diet supplementation with ATC enhances the growth performance and antioxidant properties of Hortobagy geese.

## 1. Introduction

As consumption levels increase, the safety of livestock and poultry products has received increasing attention. At present, antibiotic growth promoters have been banned in livestock and poultry production by many countries. Both the EU [1] and the US Food and Drug Administration [2] have banned the use of antibiotics as growth promoters in animals. Therefore, it is urgent to develop green and safe feed additives.

Green feed additives can help realize the environmentally friendly, high-quality development of animal husbandry, which is the foundation for the sustainable development of animal husbandry. *Acremonium terricola* culture (ATC) is a product that includes *Acremonium terricola* isolated from *Cordyceps* gunnii and is prepared by liquid submerged fermentation technology. Its main active ingredients include cordycepin, adenosine, and ergosterol, which have multiple functions such as growth promoting, antitumor [3,4], antioxidant, and immune-boosting activities [5,6]. ATC has no toxic side effects at normal doses. Adenosine is a modulator that has a universal inhibitory effect on neuronal activity. Recent studies have shown that adenosine has varied functions such as regulating sleep and wakefulness levels, neuroprotective roles, affecting susceptibility to epilepsy, and analgesic effects [7]. Cordycepin is an active ingredient of the entomopathogenic fungus *Cordyceps* and belongs to a class of compounds with significant therapeutic potential [8]. Ergosterol is a steroid compound commonly found in macrofungi, mainly in their cell membranes. Ergosterol maintains the normal metabolism of fungal cells and can enhance the body’s ability to resist diseases. Additionally, ergosterol is an important fat-soluble vitamin D_2_ source [9,10] and has obvious antibacterial and antitumor effects [11]. Cordycepin has an inhibitory effect on bacteria. It interferes with cell functions by destroying the bacterial cell membrane and combining with bacterial genomic DNA, which ultimately leads to bacterial death [12]. Cordycepin can increase antioxidant enzyme levels and inhibit neuroinflammation [13]. A hot water extract of *Cordyceps* militaris, which has a similar composition to ATC, exhibits immunomodulatory effects on broiler chickens, and can be used as a new feed additive for broiler chicken inflammation-related diseases and bacterial infections [14].

The Hortobagy goose is a high-quality Hungarian goose breed that has the characteristics of a high down content, pure white down color, large down size, minimal impurities, a good hand feel, and a high degree of fluffiness. The quality of its goose down is comparable to that of high-quality goose down produced in Hungary and Poland, and it is quite competitive in the international market. Hortobagy goose has the advantages of a short production cycle, a low cost, and a high egg production rate [15,16], thus, the Hortobagy goose has been introduced and raised in large quantities in China.

ATC is currently used in pigs [17], chickens [18], and dairy cows [19]. However, to the best of our knowledge, its use has not been reported in goose production. In this study, Hortobagy geese were selected as the experimental material, and the effects of different levels of ATC supplementation in the basal diet on the growth performance, slaughter performance, serum biochemical index, immune organ index, and serum and muscle antioxidant capacity of Hortobagy geese were studied.

## 2. Materials and Methods

### 2.1. Experimental Design, Diets and Management

The animal sample collection protocol was approved by the Animal Welfare Committee of Yangzhou University (permit number: SYXK (Su) IACUC 2012–0029). Hortobagy geese of the same batch were provided by Xiangtiange Poultry Breeding Family Farm of He County (Maanshan, China). ATC is provided by Meitedo (Jiangsu) Biotechnology Co., Ltd. (Yangzhou, China) under approval number Q/MTD321011-2020. The main active components of ATC include 350 mg/kg adenosine, 350 mg/kg cordycepin, and 550 mg/kg ergosterol. The geese were randomly assigned to 4 treatments with 6 replicates per treatment and 30 geese per replicate (half male and half female). All geese were offered the same basal diet with supplementation with 0, 3, 5, and 7 g/kg ATC. Groups were designated the control, ATC_3_, ATC_5_, and ATC_7_ groups, respectively. The composition and nutrient levels of the experimental diets are displayed in Table 1. The experiment was conducted from 1 to 70 days of age, and geese were raised on a grid with free intake of food and water. The geese were reared indoors in a goose house, and a fan was used to keep the house ventilated. For the first 2 weeks, the geese were reared in the brooding house (a layer of barbed wire was laid on the geese house, and a water pipe was laid under the barbed wire for temperature control), and the house temperature was controlled at 25~30 °C with 6 geese/m^2^. From the 3rd week, the geese were transferred to the rearing house (a layer of manure leakage board is placed on the geese house), and the house temperature was controlled at 20~25 °C with, 2 geese/m^2^.

### 2.2. Sample Collection and Measurement

During the feeding process of the experimental geese, the feed intake per day and the number of deaths were recorded in detail, and the entire experimental group was weighed on an empty stomach every week to calculate and evaluate the average daily gain (ADG), average daily feed intake (ADFI) and feed conversion ratio (FCR). ADG, ADFI, and FCR were calculated using the following formula: ADFI = Feed consumption/(Number of test days × Number of test geese); ADG = Total weight gain/(Number of test days × Number of test geese); FCR = Feed consumption/Total weight gain. An empty stomach was achieved by removing the feed from geese for 8 h. At day 70, 12 males and 12 females were randomly chosen from each treatment for slaughter sampling. The animals were fasted for 6 h before slaughtering and were weighed to record the live weight before slaughter. Before slaughter, approximately 2 mL of wing venous blood was collected and centrifuged at 3500 r/min for 10 min, and the upper serum was retained and stored in a −20 °C refrigerator for analysis of serum biochemical parameters. All geese were slaughtered by manual exsanguination. The breast and leg muscles were collected and placed in a refrigerator at −70 °C for testing. After slaughtering, the carcass weight, half evisceration weight, full evisceration weight, breast muscle weight, leg muscle weight, and abdominal fat weight were measured. Half-eviscerated weight with giblet refers to the weight of the carcass after removing the trachea, esophagus, crop, intestine, spleen, pancreas, gallbladder, reproductive organs, stomach contents, and horny membrane. Eviscerated carcass yield refers to the weight of the half evisceration weight minus the heart, liver, glandular stomach, gizzard, lungs, and abdominal fat.

### 2.3. Immune Organ Parameter Determination

On day 70, the geese were slaughtered, and the bursa of Fabricius, spleen, and liver were collected and weighed. The weight was recorded, and the immune organ index was calculated. The immune organ index is the weight of the immune organ divided by the percentage of live body weight [20]. The following formula is used for the calculation: Relative weight = Organ weight (g)/Body weight (g).

### 2.4. Serum Biochemical Parameters

Serum biochemical indicators were measured using commercial (Zhongshan Biaojia Biotechnology, Zhongshan, China.) kits and an automatic biochemical analyzer (7600, Hitachi, Tokyo, Japan). The following indicators were assessed: GLU, ALT, aspartate aminotransferase (AST), total protein (TP), alkaline phosphatase (ALP), total cholesterol (CHO), and triglyceride (TG) levels. A total protein assay kit was used to determine TP levels. An aminotransferase assay kit was used to determine AST, ALP, and ALT levels. A glucose assay kit was used to determine GLU, and enzymatic colorimetric method to determine TG and CHO levels. The procedures were performed following the kit instructions for specific detection methods.

### 2.5. Analysis of Antioxidant Status

Total antioxidant capacity (T-AOC) and malondialdehyde (MDA) levels were determined in serum and muscle using a kit (Nanjing Jiancheng Institute of Bioengineering Co., Ltd., Nanjing, China). The procedures were performed following the kit instructions for specific detection methods.

### 2.6. Statistical Analysis

The experimental data were statistically analyzed using Excel 2013 and SPSS 25.0. All the data were statistically analyzed by one-way analysis of variance (ANOVA) with SPSS 25.0 and the multi-comparison test was performed by using the least significant difference method (LSD). All the data are presented as the mean and standard error of the mean (SEM). Significant differences among all treatment means were noted when *p* < 0.05.

## 3. Results

### 3.1. Growth Performance

The effects of ATC on the growth performance of geese are shown in Table 2. Regardless of sex, ADG was not significantly different among the four treatments (*p* > 0.05). The ADFI of ATC_5_ and ATC_7_ male geese were highly significantly reduced compared with the control (*p* < 0.01). The ADFI of ATC_3_ and ATC_5_ female geese were highly significantly increased compared with that of the control (*p* < 0.01). Compared with the control group, the FCR of the Hortobagy geese was significantly reduced in the ATC_7_ group (*p* < 0.05). The FCR of ATC_3_ is less than that of ATC_5_ and ATC_7_, and the difference is highly significant (*p* < 0.01).

### 3.2. Slaughter Performance

Table 3 reveals the effects of ATC on slaughter performance. Regardless of sex, ATC supplementation of the basal diet did not significantly affect the slaughter rate, the semi-eviscerated rate, or the full-eviscerated rate of geese (*p* > 0.05). The breast and leg muscle rates of the female geese in ATC_3_ were significantly higher than those in the control (*p* < 0.05). The abdominal fat rate of ATC_5_ and ATC_7_ male geese was significantly greater than that of ATC_3_ (*p* < 0.01 or *p* < 0.05).

### 3.3. Immune Organ Parameters

Table 4 shows that the addition of ATC to the basal diet did not have a significant effect on the immune organ index of geese (*p* > 0.05).

### 3.4. Serum Biochemical Parameters

Table 5 shows the effects of ATC on the biochemical indexes of goose serum. Regardless of sex, ATC supplementation of the basal diet did not have a significant effect on the serum concentrations of ALT, ALP, CHO, TG, and TP (*p* > 0.05). Compared with the control, ATC_5_ and ATC_7_ highly significantly reduced the blood GLU concentration in the male goose serum (*p* < 0.01). ATC_3_ and ATC_5_ significantly reduced the serum ALT content in female geese (*p* < 0.05). ATC_3_ significantly reduced the average serum ALT content of male and female geese (*p* < 0.05).

### 3.5. Antioxidant Parameters

Table 6 indicates the effects of ATC on the antioxidant indexes of the geese. Compared with the control, ATC_3_ (*p* < 0.05) and ATC_5_ (*p* < 0.01) significantly or highly significantly increased the T-AOC and decreased the MDA content in the serum. The MDA content of breast muscle in the ATC_3_ and ATC_7_ groups was highly significantly decreased (*p* < 0.01), and that under ATC_5_ was significantly higher than those under ATC_3_ and ATC_7_ (*p* < 0.05). ATC3 increased the T-AOC of female geese breast muscle compared with the control (*p* < 0.01), ATC_5_ and ATC_7_ levels (*p* < 0.05). ATC_5_ highly significantly increased the T-AOC of leg muscles compared with the control (*p* < 0.01) and significantly increased the level compared with ATC_7_ (*p* < 0.05). The MDA content of the breast and leg muscles of female geese did not differ among the treatments (*p* > 0.05).

## 4. Discussion

ADG, ADFI, and FCR are the main indicators used to measure the growth performance of poultry. Li et al. [21] found that supplementation of the diet with 5 g/kg ATC improved the growth performance of weaned calves. Deng et al. [22] found that the growth performance and health of pacific white shrimp is enhanced by adding *Cordyceps* mycelial polysaccharide to feed. Supplementation of the diet with 1 g/kg *Cordyceps* militaris fermentation product positively affected the growth performance of broilers [23]. This study showed that ATC_3_ and ATC_5_ significantly increase the ADFI of Hortobagy geese. Mechanistically, ATC increases ADFI because its functional components potentially increase the appetite of geese. The addition of 7 g/kg ATC to the diet significantly reduced the FCR of Hortobagy geese. As the ATC concentration increased, both ADFI and FCR decreased significantly. Supplementation with different concentrations of ATC had no negative effects on growth performance.

Slaughter performance is the main indicator used to measure the economic benefits of breeding [24]. It is generally believed that a slaughter rate of greater than 80% and a full evisceration rate of greater than 60% are signs of good meat production performance. In this experiment, the slaughter rate of geese was 85.01–86.74%, and the eviscerated rate was 67.41–69.75%. The results showed that the meat production performance of Hortobagy geese was good. It is possible that the active ingredients in ATC, such as cordycepin, adenosine and ergosterol promote the absorption and utilization of nutrients by the body, thereby improving the slaughtering performances of geese. The specific mechanism needs further research. The supplementation of ATC in the basal diet had no adverse effect on the slaughter rate, half evisceration rate, or full evisceration rate of Hortobagy geese.

The immune organ index is an important parameter for livestock and poultry safety evaluation [25]. The immune organs of poultry mainly include the spleen, the bursa of Fabricius, and the liver. The spleen is an important peripheral immune organ. Its lymphocytes initially migrate from the central immune organ and reproduce upon antigen stimulation. Thus, the spleen has immune functions and is an important site for the immune response. The bursa of Fabricius is a unique central immune organ of poultry that can promote humoral immunity [26,27,28]. The liver has powerful phagocytic immune cells, has a stress defense ability and is also an important immune organ. The liver also has detoxification and hematopoietic functions. The immune response of the body can be evaluated by changes in the immune organ index. In this experiment, diet supplementation with ATC had no significant effect on the immune organ index of Hortobagy geese. This finding may be related to the species used in this study and the breeding environment.

Blood is an important component of the internal environment in the body and can provide a reference for animal growth and disease diagnosis [29]. Therefore, blood biochemical indicators directly reflect animal growth and metabolism. ALT and AST can reflect the permeability and metabolic capacity of liver tissues and cells [24]. Alanine aminotransferase activity is an important indicator reflecting liver function damage. When the normal function of the liver is impaired, the alanine aminotransferase activity in the blood increases [30]. This experiment showed that ATC_3_ and ATC_5_ significantly reduced the ALT content in the serum of the female geese. ATC_5_ and ATC_7_ significantly reduced the GLU content in the serum of male geese. The decrease in GLU in serum demonstrated that ATC promotes glucose metabolism. The specific metabolic pathway requires further investigation. The addition of ATC to the basal diet did not significantly affect the contents of ALP, AST, CHO, TG, and TP in goose serum.

T-AOC is a comprehensive index reflecting the body’s antioxidant system. Moreover, MDA is the product of lipid peroxidation [31,32]. MDA production increases the spatial structure of a variety of enzymes embedded in the biological cell membrane that interfere with the arrangement of the phospholipid bilayer, which subsequently causes the lipids and proteins of the bilayer to crosslink and polymerize to form lipofuscin [33,34]. As a result, the cell membrane shows extensive damage and disruption, affecting its normal function. MDA is typically used to represent the amount of free radicals produced [35]. The addition of 5 g/kg ATC to the basal diet significantly increased the T-AOC, glutathione peroxidase, and the total superoxide dismutase activities of weaned calves [21].

Regardless of gender, the T-AOC in the serum of ATC_3_ and ATC_5_ geese was highly significantly increased compared with that in the control. Compared with ATC_7_, ATC_5_ significantly increased T-AOC in serum. Compared with the control, the MDA in the serum of male geese in the ATC_3_, ATC_5_, and ATC_7_ groups was significantly reduced. The ATC_3_ and ATC_5_ groups showed significantly reduced MDA contents in the serum of female geese and significantly increased T-AOC in the muscle. ATC_3_ and ATC_7_ significantly reduce the MDA content in the breast muscles of geese. *Cordyceps* militaris polysaccharide significantly enhances the antioxidant activity of immunosuppressed mice [36]. Additionally, *Cordyceps* mycelial polysaccharides can significantly improve superoxide dismutase activity, glutathione reduction, reactive oxygen species scavenging, T-AOC, and other antioxidant indexes in prawns [22]. The results of this experiment are consistent with those of the abovementioned studies. Based on the above research results, ATC improves the body’s antioxidant capacity. The main mechanism may involve the roles of active components such as cordycepin in ATC, and the specific mechanism requires further research.

## 5. Conclusions

In conclusion, supplementation with 3 g/kg and 5 g/kg ATC enhanced the growth performance of Hortobagy geese, and 5 g/kg and 7 g/kg ATC supplementation in the basal diet increased the antioxidant capacity of Hortobagy geese. The application of ATC supplementation in geese production can improve the body’s antioxidant capacity and promote body growth, and ATC can be used as an alternative for antibiotic growth promotion. Considering the above indicators and the costs of production comprehensively, the recommended supplementation level of ATC in the basal diet of geese is 5 g/kg.

## Figures and Tables

**Table 1:** Composition and nutrient levels of the experimental diets (air-dried values).

Items	Content	
	0~2 Weeks	3~10 Weeks
Composition (g/kg)		
Corn	580	580
Soybean meal	160	50
Wheat bran	100	160
Corn gluten meal	20	20
Rice bran meal	90	160
Premix ^1^	50	30
Total	1000	1000
Nutrient levels ^2^		
ME(MJ/kg)	11.04	10.96
Crude protein (g/kg)	158.0	130.2
Crude fat (g/kg)	40.5	42.2
Crude fiber (g/kg)	40.7	43.5
Calcium (g/kg)	1.7	1.5
Available phosphorus (g/kg)	5.1	6.2
Lysine (g/kg)	7.4	5.3
Methionine (g/kg)	2.7	2.3

^1^ Premix for 0~2 weeks of age was provided per kilogram of diet: 120 KIU retinol, 24.5 KIU cholecalciferol, 500 mg a-tocopherol, 50 mg menadione, 45 mg thiamine, 185 mg riboflavin, 72 mg pyridoxine, 0.28 mg cobalamin, 900 mg niacin, 90 mg pantothenic acid, 24 mg folic acid, 3 mg biotin, 4500 mg choline chloride, 700 mg iron, 8 mg copper, 1440 mg manganese, 1100 mg zinc, 2 mg iodine, and 2 mg selenium. Premix for 3~10 weeks of age was provided per kilogram of diet: 130 KIU retinol, 19 KIU cholecalciferol, 100 mg a-tocopherol, 10 mg menadione, 26 mg thiamine, 40 mg riboflavin, 60 mg pyridoxine, 0.1 mg cobalamin, 700 mg niacin, 400 mg pantothenic acid, 12 mg folic acid, 4 mg biotin, 10,000 mg choline chloride, 1000 mg Iron, 90 mg copper, 600 mg manganese, 600 mg zinc, 8 mg iodine, and 2 mg selenium. ^2^ Metabolizable energy, crude protein, calcium, crude fat, crude fiber, lysine, methionine, and available phosphorus are calculated values.

**Table 2:** The effects of ATC on the growth performance of geese.

Item ^1^	Sex ^2^	CON	ATC_3_	ATC_5_	ATC_7_	SEM ^3^	*p*-Value
Initial BW (g)	♂	102.48	99.84	96.32	102.83	0.930	0.535
	♀	98.75	102.08	100.52	98.73	1.031	0.808
Final BW (g)	♂	3962.60	4127.36	4099.18	4226.20	65.486	0.577
	♀	3456.88	3701.58	3600.46	3464.19	36.025	0.473
ADFI (g)	♂	219.22 ^B^	245.48 ^A^	210.33 ^C^	201.68 ^D^	2.791	<0.001
	♀	185.68 ^C^	194.41 ^A^	190.81 ^B^	182.49 ^D^	0.833	<0.001
ADG (g)	♂	55.14	57.54	57.18	58.90	0.936	0.577
	♀	47.97	51.42	50.00	48.08	0.902	0.487
FCR (g/g)	♂	4.01 ^AB^	4.29 ^A^	3.72 ^BC^	3.46 ^C^	0.080	<0.001
	♀	3.89	3.82	3.88	3.85	0.075	0.988

A, B, C, and D: In the same row, values with different capital letter superscripts indicate highly significant differences (*p* < 0.01). ^1^ BW = bodyweight; ADFI = average daily feed intake; ADG = average daily gain; and FCR = feed conversion ratio. ^2^ Sex, male or female; Con, ATC_3_, ATC_5_, and ATC_7_ represent the data from geese fed 0 g, 3 g, 5 g, and 7 g ATC per kilogram of the basal diet, respectively. ^3^ SEM = standard error. *p*-value: *p*-value < 0.05 indicates a significant difference among the four groups; *p*-value < 0.01 indicates a highly significant difference between the four groups.

**Table 3:** Effects of ATC on the slaughter performance (%) of geese (*n* = 12).

Item	Sex ^1^	CON	ATC_3_	ATC_5_	ATC_7_	SEM ^2^	*p*-Value
Slaughter yield	♂	85.88	85.01	86.65	86.48	0.397	0.486
	♀	86.52	85.27	85.60	86.74	0.277	0.178
Half-eviscerated carcass yield	♂	77.70	78.14	78.58	79.23	0.341	0.457
	♀	78.75	76.70	77.85	78.14	0.399	0.279
Eviscerated carcass yield	♂	68.26	68.81	69.75	69.62	0.383	0.675
	♀	69.27	67.72	68.41	67.41	0.386	0.352
Breast yield	♂	11.31	12.17	11.46	11.58	0.164	0.289
	♀	11.47 ^Ab^	12.84 ^Aa^	11.66 ^Ab^	10.76 ^Bb^	0.236	0.006
Thigh yield	♂	14.19	15.01	15.63	14.49	0.212	0.067
	♀	14.46 ^Ab^	14.79 ^Aa^	13.67 ^Bb^	15.49 ^Aa^	0.215	0.012
Abdominal fat yield	♂	3.26 ^ab^	2.63 ^b^	3.68 ^a^	3.18 ^ab^	0.138	0.047
	♀	3.34 ^ABab^	2.75 ^Bb^	3.67 ^Aa^	3.30 ^ABa^	0.107	0.050

a, b, c, and d: In the same row, values with different small letter superscripts indicate a significant difference (*p* < 0.05). A, B, C, and D: In the same row, values with different capital letter superscripts indicate a highly significant difference (*p* < 0.01). ^1^ Sex, male or famale; CON, ATC_3_, ATC_5_, and ATC_7_ represented the data from geese fed with 0 g, 3 g, 5 g, and 7 g ATC per kilogram of the basal diet, respectively. ^2^ SEM = standard error. *p*-value: *p*-value < 0.05 indicates a significant difference among the four groups; *p*-value < 0.01 indicates a highly significant difference between the four groups.

**Table 4:** Effects of ATC on the immune organ index (%) of geese (*n* = 12).

Item	Sex ^1^	CON	ATC_3_	ATC_5_	ATC_7_	SEM ^2^	*p*-Value
Liver Index	♂	2.36	2.28	2.43	2.30	0.043	0.637
	♀	2.20	2.20	2.55	2.44	0.062	0.105
Bursal Index	♂	0.07	0.06	0.07	0.07	0.004	0.918
	♀	0.08	0.07	0.06	0.06	0.004	0.314
Spleen Index	♂	0.09	0.08	0.09	0.09	0.005	0.628
	♀	0.09	0.09	0.10	0.10	0.005	0.954

a, b, c, and d: In the same row, values with different small letter superscripts indicate a significant difference (*p* < 0.05). A, B, C, and D: In the same row, values with different capital letter superscripts indicate a highly significant difference (*p* < 0.01). ^1^ Sex, male and female; CON, ATC_3_, ATC_5_, and ATC_7_ represent the data from geese fed 0 g, 3 g, 5 g, and 7 g ATC per kilogram of the basal diet, respectively. ^2^ SEM = standard error. *p*-value: *p*-value < 0.05 indicates a significant difference among the four groups; *p*-value < 0.01 indicates a highly significant difference between the four groups.

**Table 5:** The effects of ATC on the biochemical indexes of goose serum (*n* = 12).

Item ^1^	Sex ^2^	Con	ATC_3_	ATC_5_	ATC_7_	SEM ^3^	*p*-Value
ALP (U/L)	♂	573.83	625.67	583.67	616.67	13.844	0.509
	♀	598.50	567.33	626.67	613.83	12.564	0.396
ALT (U/L)	♂	22.33	18.50	23.17	19.33	0.807	0.108
	♀	22.83 ^a^	17.83 ^b^	18.00 ^b^	21.83 ^ab^	0.772	0.025
AST (U/L)	♂	31.17	36.33	37.50	37.67	1.805	0.570
	♀	36.67	32.33	28.67	36.00	1.878	0.429
CHO (mmol/L)	♂	4.34	4.26	3.76	4.46	0.236	0.762
	♀	3.97	4.33	4.76	3.96	0.221	0.558
GLU (mmol/L)	♂	13.62 ^A^	12.56 ^AB^	11.80 ^B^	11.39 ^B^	0.264	0.007
	♀	11.57	11.38	12.04	11.33	0.221	0.678
TG (mmol/L)	♂	2.60	2.77	2.15	2.70	0.230	0.801
	♀	2.66	2.21	2.15	2.09	0.121	0.345
TP (g/L)	♂	41.50	45.37	44.08	37.67	0.779	0.307
	♀	44.03	42.38	42.60	40.75	0.600	0.301

a, b, c, and d: In the same row, values with different small letter superscripts indicate a significant difference (*p* < 0.05). A, B, C, and D: In the same row, values with different capital letter superscripts indicate a highly significant difference (*p* < 0.01). ^1^ ALP, alkaline phosphatase; ALT, alanine aminotransferase; AST, aspartate aminotransferase; CHO, total cholesterol; GLU, glucose; TG, triglyceride; TP, total protein. ^2^ Sex, male and female; CON, ATC_3_, ATC_5_, and ATC_7_ represent the data from geese fed 0 g, 3 g, 5 g, and 7 g ATC per kilogram of the basal diet, respectively. ^3^ SEM = standard error. *p*-value: *p*-value < 0.05 indicates a significant difference among the four groups; *p*-value < 0.01 indicates a highly significant difference between the four groups.

**Table 6:** The effects of ATC on the antioxidation indexes of geese (*n* = 12).

Item ^1^	Sex ^2^	CON	ATC_3_	ATC_5_	ATC_7_	SEM ^3^	*p*-Value
Serum MDA (nmol/mL)	♂	7.27 ^Aa^	6.65 ^ABb^	6.30 ^Bb^	6.69 ^ABb^	0.106	0.005
	♀	6.52 ^Aa^	6.11 ^ABb^	5.96 ^Bb^	6.30 ^AaBb^	0.070	0.027
Serum T-AOC (unit/mL)	♂	13.93 ^Bb^	15.34 ^AaB^	15.53 ^Aa^	14.62 ^AaBb^	0.212	0.018
	♀	14.09 ^Bbc^	15.33 ^ABab^	16.24 ^Aa^	14.94 ^ABbc^	0.248	0.010
Breast T-AOC (unit/mg)	♂	1.12	1.02	1.16	1.09	0.048	0.767
	♀	0.93 ^Bb^	1.32 ^Aa^	1.06 ^ABb^	1.00 ^ABb^	0.051	0.026
Breast MDA (nmol/g)	♂	13.16 ^Aa^	10.24 ^Bb^	12.45 ^ABa^	10.04 ^Bb^	0.435	0.008
	♀	11.20	9.43	9.78	9.82	0.565	0.730
Thigh T-AOC (unit/mg)	♂	1.32	1.15	1.19	1.18	0.058	0.761
	♀	0.82 ^Bb^	1.23 ^ABab^	1.48 ^Aa^	0.96 ^ABb^	0.089	0.030
Thigh MDA (nmol/g)	♂	11.60	12.00	10.23	11.27	0.432	0.547
	♀	11.68	12.63	10.30	11.49	0.392	0.218

a, b, c, and d: In the same row, values with different small letter superscripts indicate a significant difference (*p* < 0.05). A, B, C, and D: In the same row, values with different capital letter superscripts indicate a highly significant difference (*p* < 0.01). ^1^ MDA, malondialdehyde; T-AOC, total antioxidant capacity. ^2^ Sex, male or female; CON, ATC_3_, ATC_5_, and ATC_7_ represent the data from geese fed 0 g, 3 g, 5 g, and 7 g ATC per kilogram of the basal diet, respectively. ^3^ SEM = standard error. *p*-value: *p*-value < 0.05 indicates a significant difference among the four groups; *p*-value < 0.01 indicates a highly significant difference between the four groups.

## Data Availability

The raw datasets used and analyzed during the current study are available from the corresponding author on reasonable request.
